# Gender equality and disease burden in 25 EU member states, 2006–2023: a fixed-effects panel analysis

**DOI:** 10.3389/fpubh.2026.1950297

**Published:** 2026-10-01

**Authors:** Adnan Kisa, Sezer Kisa

**Affiliations:** 1 Faculty of Health Sciences and Technology, Kristiania University of Applied Sciences, Oslo, Norway; 2 Department of International Health and Sustainable Development, Tulane University, New Orleans, LA, United States; 3 Faculty of Health Sciences, Department of Nursing and Health Promotion, Oslo Metropolitan University, Oslo, Norway

**Keywords:** disability adjusted life years, disease burden, European Union, fixed effects, gender equality, Global Gender Gap Index, panel data

## Abstract

**Background:**

Gender equality is widely regarded as a social determinant of population health, but most evidence is cross-sectional or based on differences between countries. Whether within-country changes in gender equality are associated with the magnitude or composition of disease burden remains unclear, particularly in high-income settings.

**Objective:**

To examine whether within-country changes in gender equality, measured by the Global Gender Gap Index (GGGI), were associated with age-standardized all-cause disease-burden magnitude and mortality–morbidity composition in 25 EU member states from 2006 to 2023.

**Methods:**

This ecological longitudinal study used country-level panel data from 25 EU member states, yielding 450 country-year observations. The GGGI was linked to Global Burden of Disease Study 2023 estimates of age-standardized DALY, YLL, and YLD rates and the YLL/YLD ratio. The main models were two-way fixed-effects regressions with country and year fixed effects, country-clustered standard errors, and adjustment for log GDP per capita, log health expenditure per capita, and mean years of schooling. Robustness was assessed using wild cluster bootstrap-t inference, Mundlak within–between decomposition, restriction to the pre-pandemic period, and alternative education and macroeconomic specifications.

**Results:**

Within-country changes in the GGGI were not significantly associated with the two primary summary outcomes: the age-standardized total DALY rate (β = −19.1 per 0.01 increase; *p* = 0.575) or the YLL/YLD ratio (β = −0.0026; *p* = 0.296). The same null pattern was observed for YLL, YLD, and sex-specific outcomes. The conclusions were unchanged across robustness and sensitivity analyses. A negative cross-country gradient did not persist after adjustment, and within- and between-country coefficients could not be statistically distinguished.

**Conclusions:**

In high-income EU countries between 2006 and 2023, within-country changes in gender equality were not detectably associated with all-cause age-standardized disease burden or the YLL/YLD ratio. This does not imply that gender equality is unrelated to population health. Rather, broad parity indicators may be too general or slow-moving to register in all-cause burden over this time horizon. Future research should link specific gender-equality domains to disease-specific, sex-specific, health-system-sensitive, and longer-lag outcomes.

## 1 Introduction

Gender equality is increasingly recognized as a structural determinant of population health. Restrictive gender norms and unequal power relations shape exposures, health behaviors, and access to care across the life course. Previous research on gender equality, norms, and health has shown that more unequal and restrictive normative environments are associated with worse health for women and men alike, through pathways that operate across the life course and may differ by sex and gender (1, 2). More broadly, gender equality is situated within the social determinants of health agenda (3, 4). Cross-national studies have linked greater gender inequality to higher child mortality (5) and to a larger overall burden of communicable and non-communicable disease (6).

Despite this, most of the evidence base rests on cross-sectional comparisons or pooled between-country analyses that cannot separate the influence of gender equality from stable, co-varying national characteristics such as economic development, health system capacity, and educational attainment. A systematic review of high-income countries concluded that associations between gender equality and health vary by the specific outcome examined and by the gender equality measure used (7). A notable exception is the work of Veas and colleagues, who applied two-way fixed effects models to a panel of 36 OECD countries from 1990 to 2017 and found that greater gender inequality, measured by the Gender Inequality Index (GII), was associated with significantly higher premature mortality and disease burden, including years of life lost by 6.82%, disability adjusted life years by 1.50%, and years lived with disability by 2.59% per 0.1 increase in the GII (8). By contrast, an ecological study of 108 low and middle-income countries found no overall association between the GII and a double burden of disease, with at most a borderline association among women (6). Whether such associations reflect gender parity specifically, rather than the development and deprivation gradients embedded in the GII, and whether they hold within high-income countries where gender parity is high and changes slowly, remains unclear. This leaves an unresolved empirical question: whether changes in parity-based gender equality within high-income countries are reflected in all-cause disease burden and mortality–morbidity composition.

Two widely used instruments are often treated interchangeably, although they measure different constructs. The UNDP Gender Inequality Index (GII) combines reproductive health, empowerment, and labor market dimensions (9). Higher GII values indicate greater gender inequality, whereas lower values indicate less inequality. Several of these dimensions move with overall development and deprivation, so associations between the GII and health may partly reflect a country's level of development rather than the gender gap. This concern has been raised in critiques of the GII's construction (10). The World Economic Forum Global Gender Gap Index (GGGI) (11), by contrast, is designed to measure the magnitude of gender gaps in economic participation, educational attainment, health and survival, and political empowerment, rather than a country's absolute resource level. Because the GGGI focuses on parity rather than deprivation, it is better suited to asking whether gender equality itself, rather than development, is associated with population health. This distinction is central to interpreting the fixed effects association reported with the GII (8), because a within-country GII effect may partly reflect changes in development and deprivation rather than changes in parity itself.

Because this study focuses on EU member states, the EIGE Gender Equality Index (12) is an important alternative measure. The GGGI was selected because the study required a continuous annual series beginning in 2006, because it is internationally benchmarked across a consistent set of parity domains, and because it measures gender gaps rather than absolute levels of development. One measurement issue is that the composite GGGI includes a Health and Survival subindex, which partly reflects sex differences in health-related indicators. The exposure should therefore be interpreted as an overall parity index, not as a purely institutional measure unrelated to health. This point also supports the need for future analyses that separate the economic, educational, political, and health-related domains of gender equality.

Disease burden can be summarized by disability adjusted life years (DALYs), which combine years of life lost to premature mortality (YLLs) and years lived with disability (YLDs) (13). The composition of burden, meaning the balance between fatal and non-fatal health loss, is also informative and can be captured by the YLL/YLD ratio. A higher ratio indicates a more mortality-dominant profile, whereas a lower ratio indicates that non-fatal morbidity contributes relatively more. This composition has a strong sex patterning. Across the leading causes of burden, women carry proportionally more morbidity-driven burden through YLDs, whereas men carry proportionally more mortality-driven burden through YLLs (14). If gender equality is associated with reductions in premature mortality more than with changes in morbidity, this may be reflected in a lower YLL/YLD ratio. Examining burden magnitude through DALYs, YLLs, and YLDs together with burden composition through the YLL/YLD ratio, therefore, provides a richer test than examining magnitude alone.

For health systems, the relevance of gender equality lies not only in equity and rights, but also in whether structural changes in gender parity are reflected in population health needs, including premature mortality, morbidity, service demand, and the balance of burden between fatal and non-fatal conditions. The distinction between YLLs and YLDs maps onto these needs. An association between greater parity and lower YLLs would point to gains in premature mortality prevention, whereas an association concentrated in YLDs would signal changing demands for chronic disease management, rehabilitation, mental health services, and long-term care. Because health systems are increasingly judged on outcomes amenable to care rather than on access alone (15, 16), and because European health systems face shifting age structures and disease profiles (17), whether gender parity tracks the magnitude and mortality–morbidity composition of burden is directly relevant to population health monitoring and health system planning, not only to social epidemiology.

The EU is a relevant policy setting because member states share formal commitments to gender equality, health equity, and comparable population health monitoring (17, 18), while still differing in welfare state arrangements, health system organization, and the pace of gender parity change. Within this setting, gender equality may shape population health through health economic pathways, including labor market participation, income security, education, political representation, prevention uptake, occupational and behavioral risks, and access to care. Longitudinal within-country evidence on gender equality and disease burden remains scarce, particularly across high-income European countries. We address this gap using a balanced panel of 25 European Union countries from 2006 to 2023 and two-way fixed effects models that account for stable between-country heterogeneity and common annual shocks, complemented by a within-between decomposition that separates cross-country gradients from within-country change. The main contribution of this study is to examine GGGI-based gender parity rather than deprivation-based gender inequality, to separate burden magnitude from mortality–morbidity composition, and to distinguish within-country change from cross-country gradients using fixed-effects and Mundlak specifications. To our knowledge, this is the first EU panel study to examine GGGI-based gender parity in relation to both all-cause disease-burden magnitude and mortality–morbidity composition.

## 2 Aim and research questions

The aim of this study was to assess whether within-country changes in gender parity, measured by the GGGI, were associated with the magnitude and mortality–morbidity composition of age-standardized all-cause disease burden in 25 EU member states between 2006 and 2023. The analysis used burden indicators relevant to population health monitoring and health system planning. Four research questions were addressed:

RQ1. Were the within-country changes in the GGGI associated with age-standardized total DALY, YLL, and YLD rates?
RQ2. Were the within-country changes in the GGGI associated with burden composition, measured by the YLL/YLD ratio?
RQ3. Were the within-country changes in the GGGI associated with sex-specific DALY, YLL, YLD, and YLL/YLD outcomes?
RQ4. Did descriptive country mean gradients between the GGGI and disease burden persist after within-between decomposition and adjustment for country-level covariates?

## 3 Analytical framing

Prior cross-national evidence, although mixed, suggested that greater gender equality may be associated with lower all-cause disease burden and a less mortality-dominant burden profile (6–8). We therefore treated this as the expected direction of association. However, because much of the existing evidence comes from cross-sectional or between-country variation, and because country and year fixed effects identify associations from within-country change over time, we did not treat this expectation as a causal prediction. Instead, we treated both the direction and magnitude of the within-country associations as empirical questions. For robustness inference, the total DALY rate and the total YLL/YLD ratio were treated as the two primary summary outcomes because they capture disease-burden magnitude and mortality–morbidity composition, respectively. Sex-specific outcomes and the log-ratio models were treated as secondary or exploratory.

## 4 Data and methods

### 4.1 Study design, data sources, and sample

This was an ecological longitudinal study using country-level panel data, with the country-year as the unit of analysis. Disease-burden estimates were obtained from the Global Burden of Disease Study 2023 (GBD 2023) (13). Gender-equality data were taken from the Quality of Government Institute's QoG DataFinder time-series dataset, which compiles the World Economic Forum Global Gender Gap Index (11, 19).

Macroeconomic covariates were obtained from the World Bank World Development Indicators (20). These included GDP per capita, measured in purchasing-power-parity terms using constant 2021 international dollars, and current health expenditure per capita, measured in purchasing-power-parity terms using current international dollars and sourced from the World Health Organization Global Health Expenditure Database. Educational covariates, including mean years of schooling and the Education Index, were obtained from the UNDP Human Development Report database (9). The percentage of the total population aged 65 years and above was obtained from the OECD indicator for the population aged 65 years and above (18).

All data were publicly available and aggregated at the country level. For each country-year, GBD 2023 estimates of all-cause DALY, YLL, and YLD were extracted by sex and for both sexes combined as age-standardized rates per 100,000 population. These estimates were merged by country and year with the GGGI and the macroeconomic, education, and demographic covariates. The analytical dataset was restricted to countries with complete data on all analytical variables for every year from 2006 to 2023, yielding the balanced panel described below.

The analytical sample comprised 25 European Union member states, corresponding to the EU-27 excluding Cyprus and Malta. Cyprus and Malta were excluded because complete data were not available for these countries across all variables and years. Their exclusion allowed the construction of a fully balanced panel with no missing values on any analytical variable. The remaining countries were observed annually from 2006 to 2023, yielding 450 country-year observations.

### 4.2 Variables and measures

The main outcomes were age-standardized all-cause DALY, YLL, and YLD rates per 100,000 population, together with the YLL/YLD ratio. The YLL/YLD ratio was calculated as the age-standardized YLL rate divided by the age-standardized YLD rate. A higher ratio indicates a more mortality-dominant burden profile, whereas a lower ratio indicates that non-fatal morbidity contributes relatively more. Age-standardized rates (ASR) were used in the main analysis to improve comparability across countries with different age structures. Sex-specific outcomes were also examined, including female and male DALY, YLL, YLD, and YLL/YLD ratio. As a sensitivity analysis, crude all-age rates were used in place of age-standardized rates, with the percentage of the population aged 65 years and above included as a covariate to account for cross-national differences in population age structure.

The YLL/YLD ratios were calculated as follows:


$$\begin{array}{l} YLL/YLD\;{ratio}_{it}\\ =\;ASR\;YLL\;rateit\;/\;ASR\;YLD\;{rate}_{it}\\ Female\;YLL/YLD\;{ratio}_{it}\\ =\;female\;ASR\;YLL\;{rate}_{it}\;/\;female\;ASR\;YLD\;{rate}_{it}\\ Male\;YLL/YLD\;{ratio}_{it}\\ =\;male\;ASR\;YLL\;{rate}_{it}\;/\;male\;ASR\;YLD\;{rate}_{it} \end{array}$$

In these expressions, i indexes country and t indexes year.

The exposure was the overall GGGI score, which ranges from 0 to 1, with higher values indicating greater gender parity and 1 indicating parity. To aid interpretation, the index was rescaled by multiplying by 100, so that the reported coefficient corresponds to a 0.01 increase in the original GGGI. The QoG time-series also includes the four GGGI subindices: Economic Participation and Opportunity, Educational Attainment, Health and Survival, and Political Empowerment. Because the values were drawn from the harmonized QoG time-series, the 2019 observation was retained as supplied by QoG. No author interpolation, averaging, or carry-forward imputation was applied.

The main models adjusted for the natural logarithm of GDP per capita, the natural logarithm of current health expenditure per capita, and mean years of schooling. GDP per capita was used to capture macroeconomic development and material resources, while health expenditure per capita was used to capture health-system resource intensity. Mean years of schooling was used as the main education control because it is a single and transparent measure of educational attainment.

In sensitivity analyses, the Education Index was reconstructed from the UNDP Human Development Report 2025 complete time series. Following the HDI education-dimension approach, the index was calculated as the arithmetic mean of the expected years of schooling and the mean years of schooling indices. Expected years of schooling were divided by 18 and mean years of schooling by 15, with each component index capped at 1.0 before averaging. The same fixed goalposts and calculation procedure were applied to all countries and years from 2006 to 2023. Because the 2025 release recomputes the full back-series using a single current methodology, the reconstructed index is internally consistent across the panel. The percentage of the population aged 65 years and above was used only in the crude-rate sensitivity analysis. Variable definitions and sources are summarized in Table 1.

**Table 1:** Variables, operationalization, sources, and analytical role.

Variable	Operationalization	Source	Role
GGGI	Global Gender Gap Index, 0–1; scaled × 100 (per 0.01)	QoG DataFinder/WEF Global Gender Gap Index	Exposure
DALY/YLL/YLD rate	Age-standardized rate per 100,000; total and by sex	GBD 2023 (IHME)	Outcome
YLL/YLD ratio	Ratio of age-standardized YLL to YLD rate	Derived	Outcome (composition)
GDP per capita	PPP, constant 2021 international $; log-transformed	World Bank (WDI)	Main control
Health expenditure	Current health expenditure per capita, PPP (current international $); log-transformed	World Bank (WDI)/WHO GHED	Main control
Mean years of schooling	UNDP schooling indicator	UNDP HDR	Main education control
Education Index	Reconstructed from EYS and MYS using capped HDI component indices	UNDP HDR 2025	Sensitivity
Population aged 65+	% of total population	OECD	Crude-rate sensitivity only

GDP per capita was obtained from the World Bank World Development Indicators as GDP per capita, PPP, constant 2021 international dollars (indicator NY.GDP.PCAP.PP.KD). Current health expenditure per capita was obtained from the World Bank World Development Indicators as current health expenditure per capita, PPP, current international dollars (indicator SH.XPD.CHEX.PP.CD), sourced from the WHO Global Health Expenditure Database. The percentage of the population aged 65 years and above was obtained from the OECD indicator for the population aged 65 years and above (share of the population aged 65 and over), OECD Data (https://www.oecd.org/en/data/indicators/elderly-population.html).

These covariates were selected because each is plausibly related to both gender equality and population health. More affluent and more educated countries tend to have higher gender parity and lower premature mortality, while health expenditure captures differences in health-system resource intensity that may influence disease burden. These variables were therefore treated as core time-varying contextual controls and potential confounders rather than as variables of primary substantive interest. The Education Index was used only in sensitivity analyses because it is a composite education measure, whereas mean years of schooling provides a more direct and transparent control for educational attainment. Population aging was not included in the main model because the main outcomes were age-standardized; it was included only in the crude-rate sensitivity models, where differences in age structure remain part of the outcome variation. The specification was kept deliberately parsimonious because the panel included 25 countries observed over 18 years, and because institutional, educational, economic, and health-system indicators are often correlated. Adding a larger set of closely related controls could have introduced over-adjustment or absorbed part of the association between gender parity and disease burden that the analysis was designed to estimate.

### 4.3 Main statistical model

The main analysis used a two-way fixed-effects (TWFE) linear panel model:


$$\begin{array}{l} Y_{it}\;=\;\beta \cdot {GGGI}_{it}+\;X_{it}^{\prime }\gamma +\alpha_{i}+\lambda_{t}+\varepsilon_{it}, \end{array}$$

where Yit is the outcome for country *i* in year *t*, GGGI*it* is the rescaled Global Gender Gap Index, X*it* is a vector of time-varying control variables, α*i* represents country fixed effects, λ*t* represents year fixed effects, and εit is the error term. The GGGI was rescaled by multiplying the original 0–1 index by 100, so β is interpreted as the association with a 0.01 increase in the original GGGI.

Country fixed effects account for stable differences between countries, including long-standing institutional, cultural, and health-system characteristics. Year fixed effects account for shocks common to all countries in a given year, including the early phase of the COVID-19 pandemic. Fixed effects were chosen *a priori* because the aim was to estimate associations from within-country change over time rather than from cross-country differences. For this reason, a Hausman test was not used as the basis for model selection.

Standard errors were clustered by country to allow for within-country serial correlation and heteroskedasticity (21). All models were linear models fitted to continuous rates or ratios. The analyses were estimated as unweighted country-year models, giving each country equal weight. The estimand is therefore the average within-country association across the 25 EU member states, not a population-weighted association across EU residents.

### 4.4 Within–between decomposition

To distinguish within-country change from between-country differences, we estimated a Mundlak correlated random-effects specification (21, 22). In this model, each time-varying variable was separated into two parts: the country-specific mean and the deviation from that mean. For the exposure, this can be written as:


$$\begin{array}{l} {GGGI}_{it}\;=\;\left({GGGI}_{it}\;-\;mean\;{GGGI}_{i}\right)\;+\;mean\;{GGGI}_{i}, \end{array}$$

where mean GGGIi is the average GGGI for country *i* over the study period. The deviation from the country mean represents the within-country component, while the country mean represents the between-country component. The same decomposition was applied to all time-varying controls.

The within coefficient estimates the association between deviations from a country's own average GGGI and deviations in disease burden over time. The between coefficient summarizes the conditional cross-country gradient, comparing countries with different average levels of GGGI after accounting for the country means of the other covariates. The within point estimate is numerically equivalent to the fixed-effects estimate, although standard errors and confidence intervals may differ slightly because the model is estimated in a correlated random-effects framework. A Wald test was used to assess whether the within and between coefficients differed from one another.

### 4.5 Robustness and sensitivity analyses

Because the panel included only 25 country clusters, conventional cluster-robust standard errors may over-reject the null hypothesis. The two primary summary outcomes were therefore re-assessed using the wild cluster bootstrap-t procedure with Rademacher weights, imposing the null hypothesis and using 9,999 replications (23). Driscoll–Kraay standard errors were also estimated as a supplementary check for cross-sectional dependence (24), with results reported in Table S18. The main interpretation, however, was based on the country-clustered and wild-bootstrap results, because these were better aligned with the small number of country clusters.

Additional sensitivity analyses re-estimated the main models in four ways: restricting the sample to the pre-pandemic period from 2006 to 2019; using crude all-age rates with the percentage of the population aged 65 years and above included as a covariate; replacing mean years of schooling with the reconstructed Education Index; and entering GDP per capita and current health expenditure per capita separately to assess the influence of collinearity between the two economic controls.

As an exploratory analysis of burden composition, the YLL/YLD ratio was also modeled on the natural-log scale for the total population and separately by sex. This specification provides a proportional interpretation and reduces the influence of scale differences in the ratio. Statistical significance was assessed using both country-clustered standard errors and the wild cluster bootstrap-t procedure.

Analyses were conducted in Python using the statsmodels and linearmodels libraries. Statistical significance was assessed at *p* < 0.05, but interpretation emphasized effect size, direction, confidence intervals, and consistency across specifications rather than significance thresholds alone. Variance inflation factors, pairwise correlations, residual diagnostics, and data-validation checks are reported in the Supplementary material to assess multicollinearity, residual dependence, heteroskedasticity, and the internal consistency of the constructed outcomes.

## 5 Results

### 5.1 Sample characteristics and sources of variation

The balanced panel included 450 country-year observations from 25 countries over 18 years. Table 2 summarizes the distributional characteristics of the analytical variables.

**Table 2:** Descriptive statistics, 25 EU countries, 2006–2023 (*N* = 450).

Variable	Mean	SD	Minimum	Maximum
GGGI	0.738	0.045	0.650	0.875
ASR DALY rate (per 100,000)	23,283	3,919	17,771	36,221
ASR YLL rate (per 100,000)	12,413	3,925	7,572	25,066
ASR YLD rate (per 100,000)	10,870	444	10,032	13,172
ASR YLL/YLD ratio	1.14	0.37	0.67	2.26
GDP per capita, PPP (constant 2021 international $)	48,902	22,630	19,025	138,678
Health expenditure per capita, PPP (current international $)	3,537	1,846	584	8,470
Mean years of schooling	11.90	1.23	7.62	14.30
Education Index	0.852	0.055	0.675	0.958
Population aged 65+ (%)	18.28	2.65	10.76	24.19

ASR, age-standardized rate per 100,000. GDP per capita is in purchasing-power-parity terms (constant 2021 international dollars); health expenditure is current health expenditure per capita in purchasing-power-parity terms (current international dollars). Both enter the models in natural logs.

The GGGI showed moderate within-country variation, with 21.6% of its total variance occurring within countries over time. By contrast, the age-standardized mortality-related outcomes varied mainly between countries, with only about 12%−13% of their total variance occurring within countries. The full within-between decomposition is reported in Table S8.

Country-level burden patterns also varied across the 25 countries. Over 2006–2023, the mean age-standardized DALY rate ranged from 18,799.3 per 100,000 in Italy to 30,585.9 in Lithuania. DALY rates declined between 2006 and 2023 in all countries, although the size of the decline varied. The largest proportional decline occurred in Lithuania (−26.1%), whereas the smallest occurred in Greece (−2.4%). For example, the DALY rate in Germany declined from 22,133.9 to 21,185.4 per 100,000 (−4.3%), compared with a decline from 20,551.8 to 19,131.4 in Spain (−6.9%). The YLL/YLD ratio also varied across countries, with mean values over the study period ranging from 0.770 in the Netherlands to 1.773 in Latvia. Country-level summary statistics are reported in Table S20. These comparisons are descriptive and are not intended to represent country-specific effects of the GGGI.

### 5.2 Main fixed-effects models

Because all models include country and year fixed effects, the coefficients are interpreted as within-country associations over time, after accounting for stable differences between countries and annual shocks common to all countries. The predominance of between-country variation in mortality-related outcomes further supports this interpretation.

In the main age-standardized fixed-effects models, within-country changes in the GGGI showed no statistically significant association with total or sex-specific disease-burden outcomes (Table 3). For the total population, the coefficient per 0.01 increase in the GGGI was −19.1 for the DALY rate (*p* = 0.575), −27.0 for the YLL rate (*p* = 0.348), 7.9 for the YLD rate (*p* = 0.559), and −0.0026 for the YLL/YLD ratio (*p* = 0.296). The sex-specific estimates followed the same pattern. None reached statistical significance; the largest point estimate was for the male YLL rate, but this estimate was also imprecise and non-significant (β = −61.5; *p* = 0.211).

**Table 3:** Main age-standardized fixed-effects estimates for the GGGI (per 0.01 increase), country-clustered standard errors.

Outcome	Coefficient	SE	95% CI	*P*
Total DALY rate	−19.09	33.55	(−88.34, 50.16)	0.575
Total YLL rate	−27.01	28.22	(−85.25, 31.23)	0.348
Total YLD rate	7.92	13.37	(−19.68, 35.51)	0.559
Total YLL/YLD ratio	−0.0026	0.0024	(−0.0076, 0.0024)	0.296
Female DALY rate	3.91	21.70	(−40.86, 48.69)	0.858
Male DALY rate	−51.24	53.22	(−161.09, 58.61)	0.345
Female YLD rate	4.89	12.76	(−21.43, 31.22)	0.705
Male YLD rate	10.26	14.29	(−19.24, 39.76)	0.480
Female YLL rate	−0.98	16.05	(−34.11, 32.16)	0.952
Male YLL rate	−61.50	47.86	(−160.27, 37.27)	0.211
Female YLL/YLD ratio	0.0001	0.0015	(−0.0029, 0.0031)	0.941
Male YLL/YLD ratio	−0.0065	0.0041	(−0.0150, 0.0019)	0.124

All models include country and year fixed effects and adjust for ln GDP per capita, ln health expenditure per capita, and mean years of schooling. N = 450; 25 countries. Full covariate tables are provided in Table S9.

The estimated associations were modest in relation to the observed disease-burden levels. Relative to the mean age-standardized DALY rate of 23,283 per 100,000, the 95% confidence interval for the GGGI coefficient corresponded to approximately −0.38%–+0.22% per 0.01 increase in the GGGI. This interval is compatible with small decreases or increases in all-cause burden, but not with large short-term changes at this scale of exposure change. For the YLL/YLD ratio, the confidence interval also included small negative and positive values, providing no evidence of a consistent within-country shift in mortality–morbidity composition. Because the covariates were included for adjustment rather than for substantive interpretation, the results focus on the GGGI coefficients. Full covariate estimates are reported in Table S9.

### 5.3 Within–between decomposition

The country-mean correlation between the GGGI and disease burden was negative before adjustment, with *r* = −0.36 for the DALY rate and *r* = −0.38 for the YLL/YLD ratio (Table S8). In the Mundlak specification, the within-country GGGI coefficients matched the fixed-effects estimates and were not statistically significant. After adjustment for the country-level means of the covariates, the between-country coefficients were positive but also non-significant, and the Wald tests did not reject equality of the within and between components for any outcome (Table 4).

**Table 4:** Within-between Mundlak decomposition for age-standardized total outcomes.

Outcome	Component	Coefficient (95% CI)	*p*	Within = between (*p*)
Total DALY rate	Within	−19.09 (−86.69, 48.51)	0.565	0.404
	Between	66.22 (−123.65, 256.10)	0.479	
Total YLL rate	Within	−27.01 (−83.86, 29.84)	0.337	0.298
	Between	74.41 (−104.65, 253.47)	0.400	
Total YLD rate	Within	7.92 (−19.02, 34.85)	0.550	0.462
	Between	−8.18 (−41.81, 25.45)	0.620	
YLL/YLD ratio	Within	−0.0026 (−0.0075, 0.0023)	0.285	0.245
	Between	0.0078 (−0.0084, 0.0239)	0.333	

Each model includes year fixed effects and the within and between components of all time-varying controls. Standard errors are clustered by country. The within-component point estimates are numerically identical to the two-way fixed-effects estimates in Table 3; small differences in standard errors and confidence intervals reflect the correlated random-effects variance structure.

The between-country estimates should be interpreted cautiously. They are based on 25 country means, and average GGGI, GDP per capita, health expenditure, and schooling are closely related across these countries. The main purpose of the Mundlak decomposition was therefore not to give a separate substantive interpretation to the adjusted between-country coefficients, but to show that the descriptive cross-country gradients did not translate into statistically significant within-country associations over time.

### 5.4 Robustness and sensitivity

For the two primary summary outcomes, the wild cluster bootstrap-t supported the same conclusions as the country-clustered standard errors. The bootstrap *p*-value was 0.580 for the DALY rate and 0.299 for the YLL/YLD ratio, indicating that the findings did not depend on conventional cluster-robust *p*-values alone. The results were also unchanged when the analysis was restricted to the pre-pandemic period from 2006 to 2019, when crude all-age rates were used with the percentage of the population aged 65 years and above included as a covariate, when GDP per capita and current health expenditure per capita were entered separately, and when the Education Index replaced mean years of schooling (Table 5). In each sensitivity specification, the confidence intervals for both the DALY rate and the YLL/YLD ratio included zero, consistent with the main fixed-effects results (Figure 1).

**Table 5:** Robustness and sensitivity analyses for the two primary summary outcomes.

Specification	DALY β	95% CI	*p*	Ratio β	95% CI	*p*
Main ASR (country-clustered)	−19.09	(−88.3, 50.2)	0.575	−0.0026	(−0.0076, 0.0024)	0.296
Wild cluster bootstrap	—	—	0.580	—	—	0.299
Pre-pandemic, 2006–2019	40.70	(−35.6, 117.0)	0.282	0.0014	(−0.0038, 0.0065)	0.590
Crude all-age + population aged 65+	−27.92	(−111.1, 55.3)	0.495	−0.0016	(−0.0068, 0.0036)	0.523
GDP control only	−27.27	(−99.2, 44.7)	0.442	−0.0032	(−0.0090, 0.0025)	0.253
Health expenditure control only	−13.10	(−84.9, 58.7)	0.710	−0.0021	(−0.0074, 0.0033)	0.439
Education Index for MYS	−25.53	(−92.6, 41.5)	0.440	−0.0030	(−0.0085, 0.0024)	0.257

GGGI coefficients are per 0.01 increase; 95% confidence intervals are country-clustered. The wild cluster bootstrap row reports the bootstrap p-value only (no analytic CI). The crude all-age and pre-pandemic rows retain the full control set.

**Figure 1:**
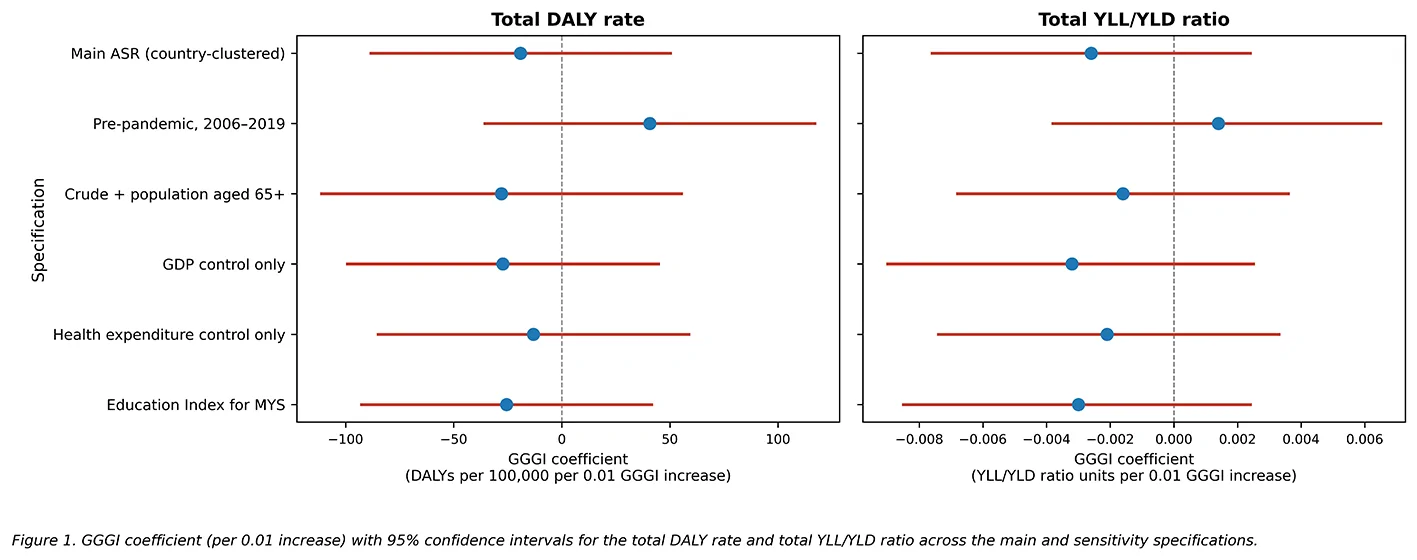
GGGI coefficient (per 0.01 increase) with 95% confidence interval for the total DALY rate and the total YLL/YLD ratio across the main and sensitivity specifications. The dashed line marks the null.

### 5.5 Exploratory composition analysis

In the exploratory log-ratio models, the GGGI was negatively associated with the male age-standardized YLL/YLD ratio (β = −0.0049 per 0.01 increase; country-clustered *p* = 0.033; wild cluster bootstrap *p* = 0.044). No comparable association was observed for the female log-ratio. The total population log-ratio showed weaker evidence of association. The corresponding male YLL/YLD ratio on the original scale was not statistically significant in the main model (Table 3). Full log-ratio results are reported in Table S17.

## 6 Discussion

In this balanced panel of 25 EU countries from 2006 to 2023, within-country changes in gender equality, measured by the GGGI, were not detectably associated with the magnitude of age-standardized all-cause disease burden or with the mortality–morbidity composition of burden measured by the YLL/YLD ratio. The null results for the two main outcomes, the total DALY rate and the YLL/YLD ratio, were robust to wild cluster bootstrap inference, exclusion of the pandemic years, crude all-age rates, an alternative education control, and alternative macroeconomic specifications. The Mundlak decomposition showed that the descriptive cross-country pattern, in which more gender-equal countries tended to have a lower burden, did not remain after adjustment for closely related indicators of economic development, health spending, and education. The within-country coefficient was also close to zero. Because the analysis included only 25 country means, the within and between components could not be statistically distinguished with high precision. The findings therefore indicate an absence of a detectable within-country association in this setting, rather than positive evidence that the relationship is only cross-sectional.

The findings showed a consistent null pattern across the four research questions. For RQ1, within-country changes in the GGGI were not detectably associated with total DALY, YLL, or YLD rates. For RQ2, they were not detectably associated with the YLL/YLD ratio on the original scale. For RQ3, the sex-specific models showed the same general pattern, with no statistically significant association in the main fixed-effects models. For RQ4, the descriptive cross-country gradient did not persist after adjustment and within–between decomposition. The expected direction of association was therefore not supported in the main within-country models. This contrasts with the significant within-country associations reported by Veas et al. using the GII, reinforcing the possibility that GII-based associations may partly reflect development gradients rather than gender parity itself.

These findings can be reconciled with the existing literature, where the association between gender equality and health is mixed and outcome-specific (7). The contrast with the most comparable study is particularly informative. Using a two-way fixed-effects panel approach, Veas and colleagues reported that greater gender inequality was associated with higher YLLs, YLDs, and DALYs across 36 OECD countries from 1990 to 2017 (8). Three differences may help explain why our study did not reproduce that pattern. First, their exposure was the GII, which combines reproductive health, empowerment, and labor market dimensions that also move with development and deprivation. Its construction has also been shown to penalize lower-income countries (10). By contrast, the GGGI measures gender parity rather than absolute development. A within-country GII effect may therefore partly capture changes in development and deprivation rather than changes in parity itself. Second, their sample covered the wider OECD, including countries with greater variation in gender inequality, whereas the present sample of EU countries occupied a narrower, high-parity range with limited within-country movement. Third, their study period was longer and ended before the COVID-19 pandemic. In our models, the total DALY and YLL point estimates were in the expected direction, with greater parity associated with lower burden, but the estimates were small and not statistically significant. The YLD estimate was positive and also not statistically significant. The fixed-effects results therefore did not support a consistent within-country association between the GGGI and all-cause disease burden.

The negative descriptive cross-country gradient also did not persist after adjustment, which is consistent with studies showing null or borderline associations between the GII and the double burden of disease in low- and middle-income countries (6). Recent cross-national evidence also shows that aggregate gender gaps in healthy life expectancy can be misleading unless their mortality and morbidity components are examined separately (25). This supports the value of examining not only the magnitude of disease burden, but also its mortality–morbidity composition.

These findings do not imply that gender equality is unrelated to population health. Rather, they suggest that in high-income EU countries, short- to medium-term changes in aggregate GGGI scores were not detectably associated with changes in all-cause age-standardized disease burden. Gender equality is one of several interacting social determinants of health (3, 4), and its health effects may operate over longer time horizons, through specific diseases, or through institutional arrangements not captured by annual aggregate fixed-effects models. The composite GGGI may also mask domain-specific mechanisms, for example, in economic participation or political empowerment, that move in different directions. All-cause age-standardized burden may further dilute pathways concentrated in particular conditions, such as cardiovascular disease, mental health, cancers detected through screening, occupational injury, substance use, interpersonal violence, and reproductive health. These are areas where gender relations are more likely to shape risk, access, and outcomes, and where effects for women and men may partly offset one another in aggregate all-cause measures.

From a health policy perspective, the findings suggest that aggregate gender-parity indices should not be expected to function as short-term predictors of all-cause disease burden in high-income EU settings. This does not reduce the importance of gender equality for health policy. Rather, it indicates that its health effects are likely to be mediated through more specific channels, including labor market participation, income security, political representation, prevention uptake, exposure to occupational and behavioral risks, and gender-sensitive access to care. Health systems seeking to reduce gendered disease burden may therefore need to monitor indicators closer to health system action than the aggregate GGGI, including cause-specific and amenable mortality (16), mental health and cardiovascular burden, screening and care access, and sex-specific care pathways (26, 27). Linking gender equity monitoring to measures of health system quality and amenable mortality (15) may better capture the pathways through which gender equality becomes relevant for population health.

At the European level, the practical implication is that gender-equality monitoring should be linked more directly to comparable health-equity indicators, including amenable mortality, screening and prevention uptake, mental health burden, cardiovascular outcomes, and sex-specific access to care. At the national level, policy responses should be guided by each country's burden profile rather than by the aggregate GGGI alone. Countries with more mortality-dominant male burden may need stronger prevention around cardiovascular disease, occupational risks, injuries, substance use, and primary-care access. Countries where morbidity accounts for a larger share of burden may need greater emphasis on mental health, chronic disease management, rehabilitation, and long-term care. These priorities are suggested by the observed burden profiles rather than derived causally from the present models.

In the exploratory log-ratio analysis, higher GGGI scores were associated with a lower mortality-dominant burden composition among men. Because men carry proportionally more mortality-driven burden than women (14), the finding in the male model is biologically and socially plausible. It could reflect sex-specific pathways, including occupational exposures, health behaviors, welfare state institutions, and healthcare access, through which gender relations shape risk (26). Consistent with a social rather than purely biological explanation, evidence from cloistered populations indicates that male excess mortality is substantially behavioral and environmental (28). At the disease-specific level, gender inequality has also been linked to higher stroke mortality (29), although our all-cause age-standardized design cannot identify such pathways. This exploratory finding should be interpreted cautiously because it emerged only in the male log-ratio model, appeared only on the log scale, was one of several secondary outcomes, and was not accompanied by a statistically significant association for the YLL/YLD ratio on the original scale. It should therefore be regarded as hypothesis-generating and requires replication using disease-specific outcomes and longer follow-up.

The null findings should also be interpreted in light of statistical precision. Although the confidence interval for the main DALY estimate rules out large short-term changes per 0.01 increase in the GGGI, it does not exclude smaller, delayed, disease-specific, or domain-specific associations. Scaled to a 0.10 change in the GGGI, the interval remains compatible with modest changes in either direction. The sign pattern of the main estimates, with a negative YLL coefficient and a positive YLD coefficient, is consistent with the possibility of mortality-to-morbidity substitution, but neither component was statistically significant, and the YLL/YLD ratio on the original scale showed no detectable shift. The exploratory male log-ratio result would also not survive correction for multiple secondary tests and should be treated as hypothesis-generating only.

### 6.1 Strengths

This study has several strengths. It uses a balanced panel of 25 EU countries with complete data from 2006 to 2023 and age-standardized GBD 2023 rates as the main outcomes, with crude all-age rates retained for sensitivity analysis. It examines both the magnitude of disease burden, measured by DALY, YLL, and YLD rates, and the mortality–morbidity composition of burden, measured by the YLL/YLD ratio. It also makes a clear conceptual and empirical distinction between the parity-based GGGI and the more deprivation-sensitive GII.

Methodologically, the two-way fixed-effects design accounts for stable between-country differences and annual shocks common to all countries. Inference is based on country-clustered standard errors and reinforced by the wild cluster bootstrap, which is appropriate given the small number of country clusters. The Mundlak within–between decomposition further separates descriptive cross-country gradients from within-country change over time. The conclusions are also supported by sensitivity analyses using crude rates, the pre-pandemic period, an alternative education control, and alternative macroeconomic specifications. Finally, the supplementary material provides a transparent audit trail, and the interpretation is deliberately cautious, avoiding causal overclaiming.

### 6.2 Limitations

Several limitations should be noted. First, the study used an ecological country-level design. The results, therefore, describe population-level associations and do not support causal or individual-level inference. Although country and year fixed effects reduce confounding from stable country characteristics and common annual shocks, reverse causality and simultaneity cannot be excluded. Changes in population health, labor-market participation, education, and political representation may influence gender-equality indicators and also affect disease burden.

Second, the analysis was based on aggregate annual measures. The composite GGGI may mask domain-specific mechanisms in economic participation, educational attainment, political empowerment, and health and survival. It also includes a Health and Survival subindex, which partly reflects health-related sex differences. This creates some potential exposure–outcome overlap when the outcomes are themselves health-burden measures. In addition, all-cause age-standardized DALY, YLL, and YLD rates may obscure disease-specific pathways, including cardiovascular disease, mental health, cancer screening, occupational injury, substance use, interpersonal violence, and reproductive health. National averages may also mask regional, socioeconomic, migrant-background, and within-country gender inequalities.

Third, the EU setting limits the range of variation. The study included 25 high-income EU countries with relatively high and slowly changing gender parity, and within-country variation in mortality-driven outcomes was limited. The 18-year study window may also be short relative to the time scale over which structural changes in gender equality affect population health. The exclusion of Cyprus and Malta improved balanced-panel comparability but limits full EU-27 representativeness. The pooled EU-25 specification may also mask heterogeneity across Nordic, Western, Southern, and Central and Eastern European welfare-state and health-system contexts, and the study was not powered to estimate reliable subregional effects.

Fourth, the models were contemporaneous and did not estimate lagged or distributed-lag associations. Delayed effects of gender-equality change, therefore, remain possible, but they were not directly tested in this study. The models also could not account for all time-varying confounders, including changes in smoking, alcohol use, obesity, migration, welfare and health-system reforms, labor-market conditions, or other public health shocks. Several secondary and exploratory analyses were conducted, including the male log-ratio analysis, which increases the risk of chance findings. The sex-specific models were also estimated separately, and formal tests of equality between male and female coefficients were not conducted.

Finally, the study used estimates and covariates from several international databases. The GBD rates were entered as point estimates, so uncertainty in the GBD estimates was not propagated through the regression models. Residual diagnostics indicated substantial serial persistence in several age-standardized outcome models (Table S19). Inference therefore relied on country-clustered standard errors, the wild cluster bootstrap, and Driscoll–Kraay estimates as a supplementary check, but the persistence of annual burden rates should be kept in mind when interpreting the estimates as adjusted within-country associations rather than causal effects. Residual measurement differences across data sources also cannot be excluded.

## 7 Conclusion

In 25 European Union countries between 2006 and 2023, within-country changes in gender equality measured by the GGGI were not detectably associated with age-standardized all-cause DALY, YLL, or YLD rates, or with the YLL/YLD ratio on the original scale. This finding was robust to wild cluster bootstrap inference and multiple sensitivity analyses. An exploratory analysis suggested an association with a less mortality-dominant burden composition in the male log-ratio model, but this result should be regarded as hypothesis-generating.

These findings suggest that aggregate annual changes in gender parity were not detectably reflected in all-cause age-standardized disease burden over this period in high-income Europe. Future research should examine disease-specific outcomes, GGGI subdomains, longer follow-up periods, broader country samples, and designs that can test mechanisms more directly. For health policy, the findings indicate that aggregate gender-parity indices should not be used as standalone indicators of short-term population-health change. Gender-equity monitoring should therefore be linked to country-specific burden profiles and to health-system-sensitive outcomes, including amenable mortality, screening and prevention uptake, access to care, mental health, cardiovascular burden, and disease-specific burden patterns.

## Data Availability

The original contributions presented in the study are included in the article/Supplementary material, further inquiries can be directed to the corresponding author.
