# Supplementary material for: Gender equality and disease burden in 25 EU member states, 2006–2023: a fixed-effects panel analysis
Source: Front Public Health. 2026 Oct 1;14:1950297. doi: 10.3389/fpubh.2026.1950297 (PMC13668557; doi:10.3389/fpubh.2026.1950297)
Supplement: Supplementary file 1 [file Table_1.docx]

**Supplementary Material**

*Gender Equality and Disease Burden in 25 EU Member States, 2006–2023: A Fixed-Effects Panel Analysis*

*This supplementary file provides the statistical audit trail supporting the main manuscript. Unless otherwise stated, estimates use the main age-standardized GBD 2023 specification. GGGI coefficients are reported per 0.01 increase in the original index. Standard errors are clustered by country, with 25 country clusters. The analytical sample includes 450 country-year observations unless otherwise noted*.

**Table S1. Countries included in the analytical panel**

**Country**

Austria

Belgium

Bulgaria

Croatia

Czechia

Denmark

Estonia

Finland

France

Germany

Greece

Hungary

Ireland

Italy

Latvia

Lithuania

Luxembourg

Netherlands

Poland

Portugal

Romania

Slovakia

Slovenia

Spain

Sweden

*Note. EU-27 excluding Cyprus and Malta.*

**Table S2. Panel structure and balance**

| **Item** | **Value** |
| --- | --- |
| Observations | 450 |
| Countries | 25 |
| Years | 18 |
| First year | 2006 |
| Last year | 2023 |
| Balanced (obs = C*T) | Yes |
| Duplicate country-year rows | 0 |
| Missing values (analytical vars) | 0 |

*Note. Cyprus and Malta were excluded because complete data were not available for them across all variables and years; all 25 included countries have complete data on every analytical variable for every year.*

**Table S3. Data validation checks for YLL/YLD ratios, DALY identities, and the Education Index**

| **Check** | **Max abs diff** |
| --- | --- |
| asr total yll yld ratio = YLL/YLD | 0.000 |
| asr total daly = YLL+YLD | 0.000 |
| asr female yll yld ratio = YLL/YLD | 0.000 |
| asr female daly = YLL+YLD | 0.000 |
| asr male yll yld ratio = YLL/YLD | 0.000 |
| asr male daly = YLL+YLD | 0.000 |
| crude total yll yld ratio = YLL/YLD | 0.000 |
| crude total daly = YLL+YLD | 0.001 |
| crude female yll yld ratio = YLL/YLD | 0.000 |
| crude female daly = YLL+YLD | 0.001 |
| crude male yll yld ratio = YLL/YLD | 0.000 |
| crude male daly = YLL+YLD | 0.001 |
| education index = mean(min(EYS/18,1), min(MYS/15,1)) | 0.000 |

*Note. Values close to zero confirm the YLL/YLD ratios, the DALY = YLL + YLD identities, and the reconstructed Education Index formula, allowing for rounding in the crude-rate estimates.*

**Table S4. Descriptive statistics for the full variable set**

| **Variable** | **Mean** | **SD** | **Min** | **Max** |
| --- | --- | --- | --- | --- |
| asr total daly rate | 23,283.1 | 3,919.5 | 17,771.2 | 36,221.1 |
| asr total yld rate | 10,870.3 | 444.276 | 10,031.6 | 13,172.2 |
| asr total yll rate | 12,412.9 | 3,925.2 | 7,571.6 | 25,066.3 |
| asr total yll yld ratio | 1.144 | 0.3663 | 0.6656 | 2.259 |
| asr female daly rate | 20,436.5 | 2,274.1 | 16,872.3 | 28,016.8 |
| asr male daly rate | 26,819.4 | 6,216.1 | 18,954.2 | 48,649.7 |
| asr female yld rate | 11,560.8 | 572.372 | 10,667.9 | 14,043.4 |
| asr male yld rate | 10,197.2 | 427.067 | 9,202.7 | 12,302.4 |
| asr female yll rate | 8,875.6 | 2,369.6 | 5,566.1 | 16,185.8 |
| asr male yll rate | 16,622.3 | 6,077.7 | 9,119.5 | 37,879.5 |
| asr female yll yld ratio | 0.7723 | 0.2209 | 0.4796 | 1.441 |
| asr male yll yld ratio | 1.625 | 0.5719 | 0.8696 | 3.517 |
| crude total daly rate | 33,966.3 | 5,859.2 | 23,908.8 | 56,703.3 |
| crude total yll yld ratio | 1.426 | 0.3752 | 0.8487 | 2.605 |
| Gggi | 0.7379 | 0.0447 | 0.6498 | 0.8750 |
| gdp pc ppp | 48,902.1 | 22,630.1 | 19,024.6 | 138678 |
| health exp pc | 3,537.3 | 1,845.8 | 584.290 | 8,469.7 |
| mean years schooling | 11.902 | 1.231 | 7.620 | 14.296 |
| education index | 0.8521 | 0.0554 | 0.6748 | 0.9583 |
| age65 pct | 18.276 | 2.653 | 10.764 | 24.191 |

*Note. ASR = age-standardized rate per 100,000 population.*

**Table S5. Variance inflation factors for the main age-standardized control set**

| **Variable** | **VIF** |
| --- | --- |
| gggi 01 | 1.627 |
| ln gdp pc ppp | 4.251 |
| ln health exp pc | 5.004 |
| mean years schooling | 1.141 |

*Note. The intercept row is omitted. Its VIF is a software artifact and is not interpreted. All substantive predictors have low to moderate VIFs.*

**Table S6. Variance inflation factors for the crude-rate control set with population aged 65 years and older**

| **Variable** | **VIF** |
| --- | --- |
| gggi 01 | 1.632 |
| ln gdp pc ppp | 6.768 |
| ln health exp pc | 7.512 |
| mean years schooling | 1.142 |
| age65 pct | 1.690 |

*Note. The intercept row is omitted. Its VIF is a software artifact and is not interpreted. All substantive predictors have low to moderate VIFs.*

**Table S7. Pairwise correlation matrix at the country-year level**

| **Variable** | **gggi 01** | **asr total daly rate** | **asr total yll rate** | **asr total yld rate** | **asr total yll yld ratio** | **ln gdp pc ppp** | **ln health exp pc** | **mean years schooling** | **age65 pct** |
| --- | --- | --- | --- | --- | --- | --- | --- | --- | --- |
| gggi 01 | 1 | -0.3750 | -0.3970 | 0.1980 | -0.4130 | 0.4410 | 0.5670 | 0.3210 | 0.2130 |
| asr total daly rate | -0.3750 | 1 | 0.9940 | 0.0440 | 0.9770 | -0.7010 | -0.8010 | 0.1460 | -0.1680 |
| asr total yll rate | -0.3970 | 0.9940 | 1 | -0.0690 | 0.9950 | -0.7190 | -0.8330 | 0.1200 | -0.1710 |
| asr total yld rate | 0.1980 | 0.0440 | -0.0690 | 1 | -0.1690 | 0.1760 | 0.2910 | 0.2290 | 0.0250 |
| asr total yll yld ratio | -0.4130 | 0.9770 | 0.9950 | -0.1690 | 1 | -0.7320 | -0.8570 | 0.0920 | -0.1750 |
| ln gdp pc ppp | 0.4410 | -0.7010 | -0.7190 | 0.1760 | -0.7320 | 1 | 0.8690 | 0.2200 | -0.1330 |
| ln health exp pc | 0.5670 | -0.8010 | -0.8330 | 0.2910 | -0.8570 | 0.8690 | 1 | 0.1890 | 0.1920 |
| mean years schooling | 0.3210 | 0.1460 | 0.1200 | 0.2290 | 0.0920 | 0.2200 | 0.1890 | 1 | 0.0020 |
| age65 pct | 0.2130 | -0.1680 | -0.1710 | 0.0250 | -0.1750 | -0.1330 | 0.1920 | 0.0020 | 1 |

*Note. Values are Pearson correlations using country-year observations.*

**Table S8. Within- and between-country variation**

| **Variable** | **Overall SD** | **Between SD** | **Within SD** | **Within %** |
| --- | --- | --- | --- | --- |
| gggi | 0.0447 | 0.0404 | 0.0208 | 21.577 |
| asr total daly rate | 3,919.5 | 3,730.2 | 1,405.3 | 12.855 |
| asr total yld rate | 444.276 | 385.345 | 233.479 | 27.618 |
| asr total yll rate | 3,925.2 | 3,745.4 | 1,382 | 12.396 |
| asr total yll yld ratio | 0.3663 | 0.3504 | 0.1267 | 11.953 |
| crude total daly rate | 5,859.2 | 5,776.7 | 1,491.1 | 6.476 |
| crude total yll yld ratio | 0.3752 | 0.3687 | 0.0997 | 7.064 |
| mean years schooling | 1.231 | 1.184 | 0.4086 | 11.014 |
| education index | 0.0554 | 0.0513 | 0.0230 | 17.298 |
| age65 pct | 2.653 | 2.142 | 1.620 | 37.294 |

*Note. Within share is the within-country variance as a percentage of total variance. Country-mean Pearson correlations between the GGGI and the total outcomes were: DALY r = −0.36, YLL r = −0.37, YLD r = +0.16, and YLL/YLD ratio r = −0.38, based on 25 country means.*

**Table S9. Main age-standardized fixed-effects models: full covariate results**

| **Outcome** | **Variable** | **Coef.** | **SE** | **t** | **p** | **CI low** | **CI high** |
| --- | --- | --- | --- | --- | --- | --- | --- |
| asr total daly rate | gggi 01 | -19.091 | 33.555 | -0.5690 | 0.575 | -88.345 | 50.162 |
| asr total daly rate | ln gdp pc ppp | -2,221.9 | 1,486.4 | -1.495 | 0.148 | -5,289.8 | 845.938 |
| asr total daly rate | ln health exp pc | -1,423.2 | 1,335.3 | -1.066 | 0.297 | -4,179.1 | 1,332.7 |
| asr total daly rate | mean years schooling | -56.342 | 464.015 | -0.1214 | 0.904 | -1,014 | 901.338 |
| asr total yld rate | gggi 01 | 7.917 | 13.370 | 0.5921 | 0.559 | -19.677 | 35.510 |
| asr total yld rate | ln gdp pc ppp | 65.783 | 428.329 | 0.1536 | 0.879 | -818.245 | 949.810 |
| asr total yld rate | ln health exp pc | -202.052 | 260.414 | -0.7759 | 0.445 | -739.520 | 335.416 |
| asr total yld rate | mean years schooling | -1.881 | 97.471 | -0.0193 | 0.985 | -203.051 | 199.289 |
| asr total yll rate | gggi 01 | -27.008 | 28.218 | -0.9571 | 0.348 | -85.246 | 31.230 |
| asr total yll rate | ln gdp pc ppp | -2,287.7 | 1,187.3 | -1.927 | 0.066 | -4,738.2 | 162.746 |
| asr total yll rate | ln health exp pc | -1,221.1 | 1,181.9 | -1.033 | 0.312 | -3,660.5 | 1,218.2 |
| asr total yll rate | mean years schooling | -54.460 | 436.205 | -0.1248 | 0.902 | -954.743 | 845.823 |
| asr total yll yld ratio | gggi 01 | -0.0026 | 0.0024 | -1.069 | 0.296 | -0.0076 | 0.0024 |
| asr total yll yld ratio | ln gdp pc ppp | -0.2066 | 0.0899 | -2.299 | 0.030 | -0.3921 | -0.0212 |
| asr total yll yld ratio | ln health exp pc | -0.1103 | 0.0954 | -1.157 | 0.259 | -0.3072 | 0.0865 |
| asr total yll yld ratio | mean years schooling | -0.0046 | 0.0380 | -0.1216 | 0.904 | -0.0831 | 0.0738 |
| asr female daly rate | gggi 01 | 3.913 | 21.695 | 0.1804 | 0.858 | -40.864 | 48.690 |
| asr female daly rate | ln gdp pc ppp | -1,383.2 | 989.688 | -1.398 | 0.175 | -3,425.9 | 659.375 |
| asr female daly rate | ln health exp pc | -1,055.6 | 751.420 | -1.405 | 0.173 | -2,606.4 | 495.274 |
| asr female daly rate | mean years schooling | 35.823 | 250.647 | 0.1429 | 0.887 | -481.487 | 553.132 |
| asr male daly rate | gggi 01 | -51.241 | 53.224 | -0.9627 | 0.345 | -161.089 | 58.608 |
| asr male daly rate | ln gdp pc ppp | -3,250.9 | 2,239 | -1.452 | 0.160 | -7,872 | 1,370.3 |
| asr male daly rate | ln health exp pc | -1,958.4 | 2,259.9 | -0.8666 | 0.395 | -6,622.6 | 2,705.7 |
| asr male daly rate | mean years schooling | -135.250 | 760.641 | -0.1778 | 0.860 | -1,705.1 | 1,434.6 |
| asr female yld rate | gggi 01 | 4.891 | 12.755 | 0.3835 | 0.705 | -21.433 | 31.215 |
| asr female yld rate | ln gdp pc ppp | -52.813 | 484.808 | -0.1089 | 0.914 | -1,053.4 | 947.780 |
| asr female yld rate | ln health exp pc | -233.573 | 283.950 | -0.8226 | 0.419 | -819.618 | 352.471 |
| asr female yld rate | mean years schooling | -15.402 | 95.615 | -0.1611 | 0.873 | -212.741 | 181.937 |
| asr male yld rate | gggi 01 | 10.259 | 14.292 | 0.7179 | 0.480 | -19.237 | 39.756 |
| asr male yld rate | ln gdp pc ppp | 163.195 | 374.191 | 0.4361 | 0.667 | -609.098 | 935.487 |
| asr male yld rate | ln health exp pc | -155.075 | 243.945 | -0.6357 | 0.531 | -658.553 | 348.404 |
| asr male yld rate | mean years schooling | 4.896 | 102.538 | 0.0478 | 0.962 | -206.731 | 216.524 |
| asr female yll rate | gggi 01 | -0.9782 | 16.054 | -0.0609 | 0.952 | -34.111 | 32.155 |
| asr female yll rate | ln gdp pc ppp | -1,330.4 | 648.077 | -2.053 | 0.051 | -2,668 | 7.139 |
| asr female yll rate | ln health exp pc | -822.007 | 575.461 | -1.428 | 0.166 | -2,009.7 | 365.686 |
| asr female yll rate | mean years schooling | 51.225 | 227.311 | 0.2254 | 0.824 | -417.922 | 520.371 |
| asr male yll rate | gggi 01 | -61.500 | 47.855 | -1.285 | 0.211 | -160.268 | 37.268 |
| asr male yll rate | ln gdp pc ppp | -3,414.1 | 1,961.6 | -1.740 | 0.095 | -7,462.6 | 634.470 |
| asr male yll rate | ln health exp pc | -1,803.4 | 2,104.8 | -0.8568 | 0.400 | -6,147.5 | 2,540.8 |
| asr male yll rate | mean years schooling | -140.146 | 726.608 | -0.1929 | 0.849 | -1,639.8 | 1,359.5 |
| asr female yll yld ratio | gggi 01 | 0.0001 | 0.0015 | 0.0746 | 0.941 | -0.0029 | 0.0031 |
| asr female yll yld ratio | ln gdp pc ppp | -0.1146 | 0.0499 | -2.296 | 0.031 | -0.2177 | -0.0116 |
| asr female yll yld ratio | ln health exp pc | -0.0800 | 0.0449 | -1.782 | 0.087 | -0.1726 | 0.0127 |
| asr female yll yld ratio | mean years schooling | 0.0053 | 0.0200 | 0.2631 | 0.795 | -0.0360 | 0.0465 |
| asr male yll yld ratio | gggi 01 | -0.0065 | 0.0041 | -1.593 | 0.124 | -0.0150 | 0.0019 |
| asr male yll yld ratio | ln gdp pc ppp | -0.3242 | 0.1460 | -2.221 | 0.036 | -0.6255 | -0.0229 |
| asr male yll yld ratio | ln health exp pc | -0.1441 | 0.1734 | -0.8311 | 0.414 | -0.5019 | 0.2137 |
| asr male yll yld ratio | mean years schooling | -0.0112 | 0.0632 | -0.1766 | 0.861 | -0.1417 | 0.1194 |

*Note. All models include country and year fixed effects. Standard errors are clustered by country. GGGI coefficients are reported per 0.01 increase in the original index.*

**Table S10. Age-standardized models additionally adjusted for population aged 65 years and older: GGGI coefficients for all outcomes**

| **Outcome** | **Variable** | **Coef.** | **SE** | **t** | **p** | **CI low** | **CI high** |
| --- | --- | --- | --- | --- | --- | --- | --- |
| asr total daly rate | gggi 01 | -16.433 | 32.170 | -0.5108 | 0.614 | -82.829 | 49.964 |
| asr total yld rate | gggi 01 | 5.594 | 12.399 | 0.4512 | 0.656 | -19.997 | 31.185 |
| asr total yll rate | gggi 01 | -22.027 | 25.712 | -0.8567 | 0.400 | -75.093 | 31.039 |
| asr total yll yld ratio | gggi 01 | -0.0020 | 0.0020 | -0.9806 | 0.337 | -0.0062 | 0.0022 |
| asr female daly rate | gggi 01 | 4.345 | 21.701 | 0.2002 | 0.843 | -40.445 | 49.134 |
| asr male daly rate | gggi 01 | -45.374 | 49.650 | -0.9139 | 0.370 | -147.848 | 57.099 |
| asr female yld rate | gggi 01 | 2.563 | 12.112 | 0.2116 | 0.834 | -22.436 | 27.561 |
| asr male yld rate | gggi 01 | 7.847 | 12.954 | 0.6057 | 0.550 | -18.889 | 34.582 |
| asr female yll rate | gggi 01 | 1.782 | 15.141 | 0.1177 | 0.907 | -29.468 | 33.031 |
| asr male yll rate | gggi 01 | -53.221 | 43.279 | -1.230 | 0.231 | -142.543 | 36.102 |
| asr female yll yld ratio | gggi 01 | 0.0004 | 0.0013 | 0.3476 | 0.731 | -0.0022 | 0.0031 |
| asr male yll yld ratio | gggi 01 | -0.0055 | 0.0035 | -1.589 | 0.125 | -0.0127 | 0.0017 |

*Note. All models include country and year fixed effects and the main control set, with population aged 65 years and older added as an additional covariate. Standard errors are clustered by country. GGGI coefficients are reported per 0.01 increase in the original index.*

**Table S11. Crude all-age models adjusted for population aged 65 years and older: GGGI coefficients for all outcomes**

| **Outcome** | **Variable** | **Coef.** | **SE** | **t** | **p** | **CI low** | **CI high** |
| --- | --- | --- | --- | --- | --- | --- | --- |
| crude total daly rate | gggi 01 | -27.916 | 40.306 | -0.6926 | 0.495 | -111.104 | 55.271 |
| crude total yld rate | gggi 01 | 5.246 | 11.639 | 0.4508 | 0.656 | -18.775 | 29.267 |
| crude total yll rate | gggi 01 | -33.163 | 37.641 | -0.8810 | 0.387 | -110.849 | 44.524 |
| crude total yll yld ratio | gggi 01 | -0.0016 | 0.0025 | -0.6480 | 0.523 | -0.0068 | 0.0036 |
| crude female daly rate | gggi 01 | -7.097 | 31.686 | -0.2240 | 0.825 | -72.494 | 58.300 |
| crude male daly rate | gggi 01 | -52.707 | 57.669 | -0.9140 | 0.370 | -171.730 | 66.315 |
| crude female yld rate | gggi 01 | 0.8448 | 10.871 | 0.0777 | 0.939 | -21.593 | 23.282 |
| crude male yld rate | gggi 01 | 9.680 | 12.743 | 0.7596 | 0.455 | -16.621 | 35.981 |
| crude female yll rate | gggi 01 | -7.942 | 30.332 | -0.2618 | 0.796 | -70.544 | 54.660 |
| crude male yll rate | gggi 01 | -62.387 | 53.770 | -1.160 | 0.257 | -173.363 | 48.588 |
| crude female yll yld ratio | gggi 01 | 0.0004 | 0.0020 | 0.2244 | 0.824 | -0.0036 | 0.0045 |
| crude male yll yld ratio | gggi 01 | -0.0047 | 0.0039 | -1.201 | 0.241 | -0.0128 | 0.0034 |

*Note. Crude all-age models include country and year fixed effects, the main control set, and population aged 65 years and older. Standard errors are clustered by country. GGGI coefficients are reported per 0.01 increase in the original index.*

**Table S12. Education Index sensitivity analysis: GGGI coefficients for all outcomes**

| **Outcome** | **Variable** | **Coef.** | **SE** | **t** | **p** | **CI low** | **CI high** |
| --- | --- | --- | --- | --- | --- | --- | --- |
| asr total daly rate | gggi 01 | -25.533 | 32.497 | -0.7857 | 0.440 | -92.604 | 41.538 |
| asr total yld rate | gggi 01 | 7.186 | 14.876 | 0.4830 | 0.633 | -23.517 | 37.889 |
| asr total yll rate | gggi 01 | -32.719 | 28.093 | -1.165 | 0.256 | -90.701 | 25.263 |
| asr total yll yld ratio | gggi 01 | -0.0030 | 0.0026 | -1.162 | 0.257 | -0.0085 | 0.0024 |
| asr female daly rate | gggi 01 | 2.560 | 20.737 | 0.1234 | 0.903 | -40.240 | 45.359 |
| asr male daly rate | gggi 01 | -62.960 | 52.040 | -1.210 | 0.238 | -170.365 | 44.445 |
| asr female yld rate | gggi 01 | 3.713 | 13.841 | 0.2682 | 0.791 | -24.855 | 32.280 |
| asr male yld rate | gggi 01 | 9.779 | 16.287 | 0.6005 | 0.554 | -23.834 | 43.393 |
| asr female yll rate | gggi 01 | -1.153 | 15.648 | -0.0737 | 0.942 | -33.450 | 31.144 |
| asr male yll rate | gggi 01 | -72.739 | 47.573 | -1.529 | 0.139 | -170.925 | 25.446 |
| asr female yll yld ratio | gggi 01 | 0.0002 | 0.0015 | 0.1036 | 0.918 | -0.0030 | 0.0033 |
| asr male yll yld ratio | gggi 01 | -0.0074 | 0.0044 | -1.704 | 0.101 | -0.0164 | 0.0016 |

*Note. Mean years of schooling is replaced by the reconstructed Education Index. All models include country and year fixed effects and the remaining main controls. Standard errors are clustered by country. GGGI coefficients are reported per 0.01 increase in the original index.*

**Table S13. Pre-pandemic period, 2006–2019: GGGI coefficients for all outcomes**

| **Outcome** | **Variable** | **Coef.** | **SE** | **t** | **p** | **CI low** | **CI high** |
| --- | --- | --- | --- | --- | --- | --- | --- |
| asr total daly rate | gggi 01 | 40.696 | 36.948 | 1.101 | 0.282 | -35.560 | 116.952 |
| asr total yld rate | gggi 01 | 13.411 | 15.140 | 0.8858 | 0.385 | -17.836 | 44.658 |
| asr total yll rate | gggi 01 | 27.285 | 29.954 | 0.9109 | 0.371 | -34.538 | 89.108 |
| asr total yll yld ratio | gggi 01 | 0.0014 | 0.0025 | 0.5463 | 0.590 | -0.0038 | 0.0065 |
| asr female daly rate | gggi 01 | 27.664 | 24.718 | 1.119 | 0.274 | -23.351 | 78.679 |
| asr male daly rate | gggi 01 | 55.468 | 55.078 | 1.007 | 0.324 | -58.206 | 169.143 |
| asr female yld rate | gggi 01 | 9.825 | 13.709 | 0.7167 | 0.480 | -18.468 | 38.118 |
| asr male yld rate | gggi 01 | 16.483 | 16.680 | 0.9882 | 0.333 | -17.943 | 50.909 |
| asr female yll rate | gggi 01 | 17.839 | 17.096 | 1.044 | 0.307 | -17.444 | 53.123 |
| asr male yll rate | gggi 01 | 38.985 | 48.467 | 0.8044 | 0.429 | -61.045 | 139.015 |
| asr female yll yld ratio | gggi 01 | 0.0010 | 0.0015 | 0.7012 | 0.490 | -0.0020 | 0.0040 |
| asr male yll yld ratio | gggi 01 | 0.0016 | 0.0040 | 0.3924 | 0.698 | -0.0067 | 0.0098 |

*Note. Models are restricted to the pre-pandemic period, 2006–2019. All models include country and year fixed effects and the main control set. Standard errors are clustered by country. GGGI coefficients are reported per 0.01 increase in the original index.*

**Table S14. Alternative macroeconomic control specifications: GGGI coefficients for the primary outcomes**

| **Model** | **Outcome** | **Coef.** | **SE** | **p** | **CI low** | **CI high** |
| --- | --- | --- | --- | --- | --- | --- |
| GDP only | asr total daly rate | -27.268 | 34.850 | 0.442 | -99.196 | 44.659 |
| GDP only | asr total yll yld ratio | -0.0032 | 0.0028 | 0.253 | -0.0090 | 0.0025 |
| Health only | asr total daly rate | -13.101 | 34.806 | 0.710 | -84.936 | 58.735 |
| Health only | asr total yll yld ratio | -0.0021 | 0.0026 | 0.439 | -0.0074 | 0.0033 |
| GDP+health (main) | asr total daly rate | -19.091 | 33.555 | 0.575 | -88.345 | 50.162 |
| GDP+health (main) | asr total yll yld ratio | -0.0026 | 0.0024 | 0.296 | -0.0076 | 0.0024 |

*Note. Models compare alternative macroeconomic control specifications while retaining country and year fixed effects and mean years of schooling. Standard errors are clustered by country. GGGI coefficients are reported per 0.01 increase in the original index.*

**Table S15. Mundlak within–between decomposition for total age-standardized outcomes**

| **Outcome** | **Component** | **Coef.** | **SE** | **p** | **CI low** | **CI high** | **W=B p** |
| --- | --- | --- | --- | --- | --- | --- | --- |
| asr total daly rate | within | -19.091 | 32.754 | 0.565 | -86.692 | 48.509 | 0.404 |
| asr total daly rate | between | 66.225 | 91.996 | 0.479 | -123.646 | 256.096 | 0.404 |
| asr total yld rate | within | 7.917 | 13.050 | 0.550 | -19.018 | 34.851 | 0.462 |
| asr total yld rate | between | -8.182 | 16.296 | 0.620 | -41.814 | 25.451 | 0.462 |
| asr total yll rate | within | -27.008 | 27.544 | 0.337 | -83.856 | 29.840 | 0.298 |
| asr total yll rate | between | 74.406 | 86.758 | 0.400 | -104.653 | 253.466 | 0.298 |
| asr total yll yld ratio | within | -0.0026 | 0.0024 | 0.284 | -0.0075 | 0.0023 | 0.245 |
| asr total yll yld ratio | between | 0.0078 | 0.0078 | 0.333 | -0.0084 | 0.0239 | 0.245 |

*Note. Each model includes year fixed effects and the within and between components of all time-varying controls. Standard errors are clustered by country. W = B p is the Wald test of equality between the within and between GGGI coefficients.*

**Table S16. Wild cluster bootstrap-t inference for the primary summary outcomes**

| **Outcome** | **Coef.** | **Cluster SE** | **t** | **Cluster p** | **Bootstrap p** |
| --- | --- | --- | --- | --- | --- |
| asr total daly rate | -19.091 | 33.555 | -0.5690 | 0.575 | 0.580 |
| asr total yll yld ratio | -0.0026 | 0.0024 | -1.069 | 0.296 | 0.299 |

*Note. Wild cluster bootstrap-t inference used Rademacher weights, imposed the null hypothesis, and used 9,999 replications. Cluster p-values are based on country-clustered standard errors.*

**Table S17. Exploratory log-transformed YLL/YLD ratio models**

| **Outcome** | **Coef.** | **Clustered p** | **Bootstrap p** | **Driscoll–Kraay p** |
| --- | --- | --- | --- | --- |
| ln asr total yll yld ratio | -0.0036 | 0.088 | 0.098 | 0.009 |
| ln asr female yll yld ratio | -0.0018 | 0.364 | 0.359 | 0.107 |
| ln asr male yll yld ratio | -0.0049 | 0.033 | 0.044 | 0.004 |

*Note. Clustered and wild cluster bootstrap p-values are the basis for interpretation. Driscoll–Kraay p-values are shown as supplementary checks. Bootstrap replications = 9,999.*

**Table S18. Driscoll–Kraay standard errors for total age-standardized outcomes**

| **Outcome** | **Coef.** | **SE** | **t** | **p** |
| --- | --- | --- | --- | --- |
| asr total daly rate | -19.091 | 31.738 | -0.6015 | 0.548 |
| asr total yld rate | 7.917 | 4.998 | 1.584 | 0.114 |
| asr total yll rate | -27.008 | 29.229 | -0.9240 | 0.356 |
| asr total yll yld ratio | -0.0026 | 0.0023 | -1.144 | 0.253 |

*Note. Driscoll–Kraay standard errors use a Bartlett kernel and are reported as a supplementary check for cross-sectional dependence.*

**Table S19. Residual diagnostics for the main age-standardized models**

| **Outcome** | **AR(1) coef** | **AR(1) p** | **BP p** | **Pesaran CD** | **CD p** |
| --- | --- | --- | --- | --- | --- |
| asr total daly rate | 0.6730 | 0.000 | 0.000 | -0.7381 | 0.460 |
| asr total yld rate | 0.9291 | 0.000 | 0.000 | -1.017 | 0.309 |
| asr total yll rate | 0.6627 | 0.000 | 0.000 | 0.1019 | 0.919 |
| asr total yll yld ratio | 0.6758 | 0.000 | 0.000 | -0.4993 | 0.618 |
| asr female daly rate | 0.5800 | 0.000 | 0.056 | -2.026 | 0.043 |
| asr male daly rate | 0.7287 | 0.000 | 0.000 | 0.5060 | 0.613 |
| asr female yld rate | 0.9284 | 0.000 | 0.000 | -2.048 | 0.041 |
| asr male yld rate | 0.9283 | 0.000 | 0.001 | 0.8927 | 0.372 |
| asr female yll rate | 0.5608 | 0.000 | 0.041 | -1.224 | 0.221 |
| asr male yll rate | 0.7203 | 0.000 | 0.000 | 1.213 | 0.225 |
| asr female yll yld ratio | 0.6033 | 0.000 | 0.017 | -1.613 | 0.107 |
| asr male yll yld ratio | 0.7153 | 0.000 | 0.000 | 0.3926 | 0.695 |

*Note. AR(1) = first-order residual autocorrelation; BP = Breusch–Pagan test; Pesaran CD = cross-sectional dependence test. These diagnostics support the use of country-clustered inference, wild cluster bootstrap checks for the primary outcomes, and Driscoll–Kraay estimates as supplementary checks.*

**Table S20. Country-level summary statistics for the main exposure and primary outcomes, 25 EU countries, 2006–2023**

| **Country** | **Mean GGGI** | **Mean ASR DALY** | **ASR DALY 2006** | **ASR DALY 2023** | **Absolute DALY change** | **DALY % change** | **Mean YLL/YLD** | **YLL/YLD 2006** | **YLL/YLD 2023** | **YLL/YLD change** |
| --- | --- | --- | --- | --- | --- | --- | --- | --- | --- | --- |
| Austria | 0.730 | 20,647.1 | 22,035.5 | 19,994.2 | -2,041.2 | -9.3% | 0.916 | 1.044 | 0.799 | -0.245 |
| Belgium | 0.756 | 21,390.7 | 22,625.3 | 20,221.7 | -2,403.6 | -10.6% | 0.928 | 1.066 | 0.763 | -0.303 |
| Bulgaria | 0.725 | 29,374.7 | 32,329.6 | 27,168.5 | -5,161.1 | -16.0% | 1.754 | 2.028 | 1.507 | -0.521 |
| Croatia | 0.712 | 24,734.2 | 26,842.0 | 23,481.8 | -3,360.2 | -12.5% | 1.302 | 1.515 | 1.113 | -0.402 |
| Czechia | 0.687 | 23,745.3 | 25,758.0 | 22,058.5 | -3,699.5 | -14.4% | 1.148 | 1.331 | 0.967 | -0.363 |
| Denmark | 0.773 | 21,201.4 | 23,262.8 | 20,285.1 | -2,977.6 | -12.8% | 0.971 | 1.214 | 0.811 | -0.403 |
| Estonia | 0.727 | 26,238.3 | 31,769.2 | 23,702.6 | -8,066.6 | -25.4% | 1.412 | 1.892 | 1.134 | -0.757 |
| Finland | 0.839 | 21,061.6 | 22,899.5 | 20,398.3 | -2,501.2 | -10.9% | 0.928 | 1.088 | 0.822 | -0.265 |
| France | 0.748 | 20,257.7 | 21,334.1 | 20,288.7 | -1,045.3 | -4.9% | 0.854 | 0.984 | 0.771 | -0.213 |
| Germany | 0.774 | 21,207.2 | 22,133.9 | 21,185.4 | -948.5 | -4.3% | 0.925 | 1.027 | 0.886 | -0.141 |
| Greece | 0.686 | 21,402.9 | 22,257.2 | 21,724.6 | -532.6 | -2.4% | 0.944 | 1.048 | 0.886 | -0.162 |
| Hungary | 0.678 | 27,743.4 | 31,139.1 | 25,660.4 | -5,478.7 | -17.6% | 1.546 | 1.827 | 1.311 | -0.515 |
| Ireland | 0.787 | 20,528.2 | 22,360.7 | 19,837.9 | -2,522.8 | -11.3% | 0.870 | 1.089 | 0.702 | -0.387 |
| Italy | 0.696 | 18,799.3 | 20,003.1 | 18,674.8 | -1,328.3 | -6.6% | 0.849 | 0.968 | 0.749 | -0.219 |
| Latvia | 0.759 | 29,900.6 | 35,891.6 | 27,661.6 | -8,230.0 | -22.9% | 1.773 | 2.259 | 1.510 | -0.749 |
| Lithuania | 0.746 | 30,585.9 | 36,221.1 | 26,764.9 | -9,456.2 | -26.1% | 1.755 | 2.247 | 1.367 | -0.880 |
| Luxembourg | 0.722 | 19,496.0 | 21,482.0 | 18,742.7 | -2,739.2 | -12.8% | 0.835 | 1.045 | 0.686 | -0.358 |
| Netherlands | 0.755 | 21,949.7 | 23,961.4 | 20,968.9 | -2,992.5 | -12.5% | 0.770 | 0.846 | 0.703 | -0.143 |
| Poland | 0.713 | 25,555.6 | 27,839.5 | 23,697.4 | -4,142.0 | -14.9% | 1.387 | 1.582 | 1.157 | -0.425 |
| Portugal | 0.732 | 21,094.3 | 23,091.5 | 20,695.6 | -2,395.9 | -10.4% | 0.926 | 1.116 | 0.790 | -0.326 |
| Romania | 0.696 | 28,952.2 | 32,264.5 | 26,531.7 | -5,732.8 | -17.8% | 1.749 | 2.035 | 1.472 | -0.564 |
| Slovakia | 0.694 | 26,310.1 | 29,081.8 | 24,093.6 | -4,988.2 | -17.2% | 1.450 | 1.690 | 1.210 | -0.480 |
| Slovenia | 0.743 | 21,584.0 | 24,358.5 | 20,204.8 | -4,153.7 | -17.1% | 0.979 | 1.218 | 0.810 | -0.407 |
| Spain | 0.755 | 19,154.3 | 20,551.8 | 19,131.4 | -1,420.4 | -6.9% | 0.823 | 0.987 | 0.726 | -0.261 |
| Sweden | 0.816 | 19,163.9 | 19,974.1 | 18,947.8 | -1,026.2 | -5.1% | 0.816 | 0.971 | 0.666 | -0.305 |

*Note. ASR = age-standardized rate per 100,000 population; DALY = disability-adjusted life year; YLL = years of life lost; YLD = years lived with disability; GGGI = Global Gender Gap Index, original 0–1 scale. Each row summarizes 18 annual observations from 2006 to 2023. Absolute and percentage changes compare 2023 with 2006 and were calculated using the unrounded values. Country-level values are descriptive and should not be interpreted as country-specific effects of the GGGI.*
